# Magnetic nanoparticles: preparation, physical properties, and applications in biomedicine

**DOI:** 10.1186/1556-276X-7-144

**Published:** 2012-02-21

**Authors:** Abolfazl Akbarzadeh, Mohamad Samiei, Soodabeh Davaran

**Affiliations:** 1 Faculty of Pharmacy, Department of Medicinal Chemistry and Drug Applied Research Center Tabriz University of Medical Sciences, Tabriz, 51368, Iran; 2 Faculity of Dentistry, Tabriz University of Medical Sciences, Tabriz, 51368, Iran; 3 Research Center for Pharmaceutical Nanotechnology, Tabriz University of Medical Sciences, Tabriz, 51368, Iran

**Keywords:** magnetic nanoparticles, synthetic routes, biomedical applications, functionalization techniques, characterization

## Abstract

Finally, we have addressed some relevant findings on the importance of having well-defined synthetic strategies developed for the generation of MNPs, with a focus on particle formation mechanism and recent modifications made on the preparation of monodisperse samples of relatively large quantities not only with similar physical features, but also with similar crystallochemical characteristics. Then, different methodologies for the functionalization of the prepared MNPs together with the characterization techniques are explained. Theorical views on the magnetism of nanoparticles are considered.

## Introduction

Nanoscience is one of the most important research in modern science. Nanotechnology is beginning to allow scientists, engineers, chemists, and physicians to work at the molecular and cellular levels to produce important advances in the life sciences and healthcare. The use of nanoparticle [NP] materials offers major advantages due to their unique size and physicochemical properties. Because of the widespread applications of magnetic nanoparticles [MNPs] in biotechnology, biomedical, material science, engineering, and environmental areas, much attention has been paid to the synthesis of different kinds of MNPs [1-3].

Real uses of nanostructured materials in life sciences are uncommon at the present time. However, the excellent properties of these materials provide a very promising future for their use in this field [4-7]. Nanoclusters are ultrafine particles of nanometer dimensions located between molecules and microscopic structures (micron size). Viewed as materials, they are so small that they exhibit characteristics that are not observed in larger structures (even 100 nm); viewed as molecules, they are so large that they provide access to realms of quantum behavior that are not otherwise accessible. In this size, many recent advances have been made in biology, chemistry, and physics [8-11]. The preparation of monodisperse-sized nanocrystals is very important because the properties of these nanocrystals depend strongly on their dimensions [12,13]. The preparation of monodisperse-sized nanocrystals with controllable sizes is very important to characterize the size-dependent physicochemical properties of nanocrystals [14-16].

Industrial applications of magnetic nanoparticles cover a broad spectrum of magnetic recording media and biomedical applications, for example, magnetic resonance contrast media and therapeutic agents in cancer treatment [17,18]. Each potential application of the magnetic nanoparticles requires having different properties. For example, in data storage applications, the particles need to have a stable, switchable magnetic state to represent bits of information that are not affected by temperature fluctuations.

For biomedical uses, the application of particles that present superparamagnetic behavior at room temperature is preferred [19-21]. Furthermore, applications in therapy and biology and medical diagnosis require the magnetic particles to be stable in water at pH 7 and in a physiological environment. The colloidal stability of this fluid will depend on the charge and surface chemistry, which give rise to both steric and coulombic repulsions and also depend on the dimensions of the particles, which should be sufficiently small so that precipitation due to gravitation forces can be avoided [22]. Additional restrictions to the possible particles could be used for biomedical applications (*in vivo *or *in vitro *applications). For *in vivo *applications, the magnetic nanoparticles must be encapsulated with a biocompatible polymer during or after the preparation process to prevent changes from the original structure, the formation of large aggregates, and biodegradation when exposed to the biological system. The nanoparticle coated with polymer will also allow binding of drugs by entrapment on the particles, adsorption, or covalent attachment [23-25]. The major factors, which determine toxicity and the biocompatibility of these materials, are the nature of the magnetically responsive components, such as magnetite, iron, nickel, and cobalt, and the final size of the particles, their core, and the coatings. Iron oxide nanoparticles such as magnetite (Fe_3_O_4_) or its oxidized form maghemite (γ-Fe_2_O_3_) are by far the most commonly employed nanoparticles for biomedical applications. Highly magnetic materials such as cobalt and nickel are susceptible to oxidation and are toxic; hence, they are of little interest [26-28]. Moreover, the major advantage of using particles of sizes smaller than 100 nm is their higher effective surface areas, lower sedimentation rates, and improved tissular diffusion [29-31]. Another advantage of using nanoparticles is that the magnetic dipole-dipole interactions are significantly reduced because they scale as r6 [32]. Therefore, for *in vivo *biomedical applications, magnetic nanoparticles must be made of a non-toxic and non-immunogenic material, with particle sizes small enough to remain in the circulation after injection and to pass through the capillary systems of organs and tissues, avoiding vessel embolism. They must also have a high magnetization so that their movement in the blood can be controlled with a magnetic field and so that they can be immobilized close to the targeted pathologic tissue [33-35]. For *in vitro *applications, composites consisting of superparamagnetic nanocrystals dispersed in submicron diamagnetic particles with long sedimentation times in the absence of a magnetic field can be used because the size restrictions are not so severe as in *in vivo *applications. The major advantage of using diamagnetic matrixes is that the superparamagnetic composites can be easily prepared with functionality.

In almost all uses, the synthesis method of the nanomaterials represents one of the most important challenges that will determine the shape, the size distribution, the particle size, the surface chemistry of the particles, and consequently their magnetic properties [36-38]. Ferri- and ferromagnetic materials such as Fe_3_O_4 _and some alloys have irregular particle shape when obtained by grinding bulk materials but can have a spherical shape when synthesized by plasma atomization, wet chemistry, or from the gas phases and aerosol. Also, depending on the mechanism of formation, spherical particles obtained in a solution can be crystalline or amorphous if they result from a disordered or ordered aggregation of crystallites, respectively. In addition, the synthesis method determines to a great extent the degree of structural defects or impurities in the particle as well as the distribution of such defects within the particle, therefore, determining its magnetic behavior [39,40]

Recently, many attempts have been made to develop techniques and processes that would yield 'monodispersed colloids' consisting of uniform nanoparticles both in size and shape [41-43]. In these systems, the entire uniform physicochemical properties directly reflect the properties of each constituent particle. Monodispersed colloids have been exploited in fundamental research and as models in the quantitative assessment of properties that depend on the particle size and shape. In addition, it has become evident that the reproducibility and quality of commercial products can be more readily achieved by starting with well-defined powders of known properties. In this way, these powders have found uses in photography, inks in printing, catalysis, ceramic, and especially in medicine.

Magnetic nanoparticles show remarkable new phenomena such as high field irreversibility, high saturation field, superparamagnetism, extra anisotropy contributions, or shifted loops after field cooling. These phenomena arise from narrow and finite-size effects and surface effects that dominate the magnetic behavior of individual nanoparticles [44]. Frenkel and Dorfman [45] were the first to predict that a particle of ferromagnetic material, below a critical particle size (*<*15 nm for the common materials), would consist of a single magnetic domain, i.e., a particle that is in a state of uniform magnetization at any field. The magnetization behavior of these particles above a certain temperature, i.e., the blocking temperature, is identical to that of atomic paramagnets (superparamagnetism) except that large susceptibilities and, thus, an extremely large moment are involved [46].

The first part of this review is concerned with the physical properties of magnetic nanoparticles and their magnetometric property. The second part deals with the possible use of magnetic nanoparticles for biomedical application with special emphasis on the advantage of using nanoparticles with respect to microparticles and on some of the recent environmental, industrial, biological, and analytical applications of MNPs. The third part deals with the different methods described in the bibliography that are capable of producing these magnetic nanoparticles with a very narrow particle size distribution, mainly based on magnetite or maghemite iron oxide nanoparticles [47,48]. Finally, we address some of the most relevant preparation effects on the magnetic properties and structure of the magnetic nanoparticles.

## Preparation Methods

During the last few years, a large portion of the published articles about MNPs have described efficient routes to attain shape-controlled, highly stable, and narrow size distribution MNPs. Up to date, several popular methods including co-precipitation, microemulsion, thermal decomposition, solvothermal, sonochemical, microwave assisted, chemical vapour deposition, combustion synthesis, carbon arc, laser pyrolysis synthesis have been reported for synthesis of MNPs. 

## Physical properties of magnetic nanoparticles

**Figure 1:**
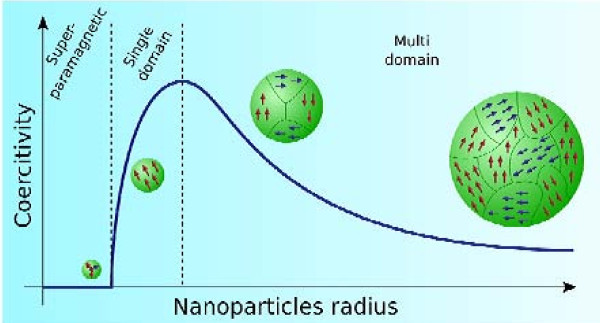
**Schematic illustration of the coercivity-size relations of small particles**.

Magnetic effects are caused by movements of particles that have both mass and electric charges. These particles are electrons, holes, protons, and positive and negative ions. A spinning electric-charged particle creates a magnetic dipole, so-called magneton. In ferromagnetic materials, magnetons are associated in groups. A magnetic domain (also called a Weiss domain) refers to a volume of ferromagnetic material in which all magnetons are aligned in the same direction by the exchange forces. This concept of domains distinguishes ferromagnetism from paramagnetism. The domain structure of a ferromagnetic material determines the size dependence of its magnetic behavior. When the size of a ferromagnetic material is reduced below a critical value, it becomes a single domain. Fine particle magnetism comes from size effects, which are based on the magnetic domain structure of ferromagnetic materials. It assumes that the state of lowest free energy of ferromagnetic particles has uniform magnetization for particles smaller than a certain critical size and has nonuniform magnetization for larger particles. The former ones are referred to as single domain particles, while the latter are called multidomain particles [49,50]. According to the magnetic domain theory, the critical size of the single domain is affected by several factors including the value of the magnetic saturation, the strength of the crystal anisotropy and exchange forces, surface or domain-wall energy, and the shape of the particles. The reaction of ferromagnetic materials on an applied field is well described by a hysteresis loop, which is characterized by two main parameters: remanence and coercivity. The latter is related to the 'thickness' of the curve. Dealing with fine particles, the coercivity is the single property of most interest, and it is strongly size-dependent. It has been found that as the particle size is reduced, the coercivity increases to a maximum and then decreases toward zero (Figure 1).

When the size of single-domain particles further decreases below a critical diameter, the coercivity becomes zero, and such particles become superparamagnetic. Superparamagnetism is caused by thermal effects. In superparamagnetic particles, thermal fluctuations are strong enough to spontaneously demagnetize a previously saturated assembly; therefore, these particles have zero coercivity and have no hysteresis. Nanoparticles become magnetic in the presence of an external magnet, but revert to a nonmagnetic state when the external magnet is removed. This avoids an 'active' behavior of the particles when there is no applied field. Introduced in the living systems, particles are 'magnetic' only in the presence of an external field, which gives them unique advantage in working in biological environments. There are a number of crystalline materials that exhibit ferromagnetism, among others Fe, Co, or Ni. Since ferrite oxide-magnetite (Fe_3_O_4_) is the most magnetic of all the naturally occurring minerals on earth, it is widely used in the form of superparamagnetic nanoparticles for all sorts of biological applications [51-53].

## Magnetic property (magnetic behavior)

**Figure 2:**
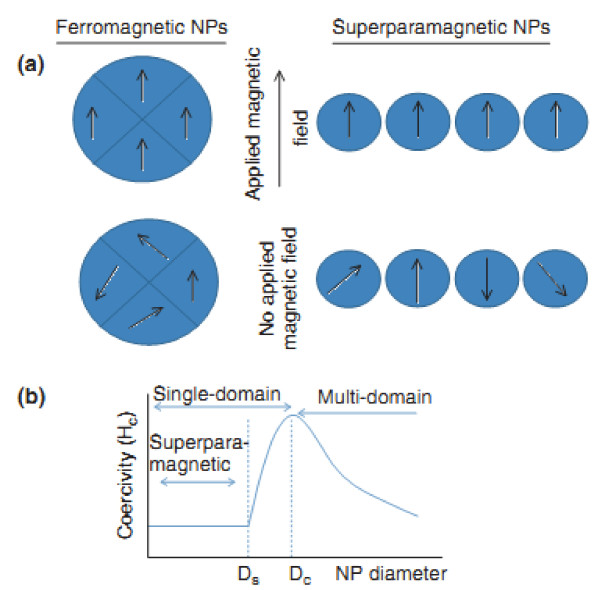
**Magnetization behavior of ferromagnetic and superparamagnetic NPs under an external magnetic field**. (**a**) Under an external magnetic field, domains of a ferromagnetic NP align with the applied field. The magnetic moment of single domain superparamagnetic NPs aligns with the applied field. In the absence of an external field, ferromagnetic NPs will maintain a net magnetization, whereas superparamagnetic NPs will exhibit no net magnetization due to rapid reversal of the magnetic moment. (**b**) Relationship between NP size and the magnetic domain structures. Ds and Dc are the 'superparamagnetism' and 'critical' size thresholds.

Materials are classified by their response to an externally applied magnetic field. Descriptions of orientations of the magnetic moments in a material help identify different forms of magnetism observed in nature. Five basic types of magnetism can be described: diamagnetism, paramagnetism, ferromagnetism, antiferromagnetism, and ferrimagnetisms. In the presence of an externally applied magnetic field, the atomic current loops created by the orbital motion of electrons respond to oppose the applied field. All materials display this type of weak repulsion to a magnetic field known as diamagnetism. However, diamagnetism is very weak, and therefore, any other form of magnetic behavior that a material may possess usually overpowers the effects of the current loops. In terms of the electronic configuration of the materials, diamagnetism is observed in materials with filled electronic subshells where the magnetic moments are paired and overall cancel each other. Diamagnetic materials have a negative susceptibility (*χ *< 0) and weakly repel an applied magnetic field (e.g., quartz SiO_2_). The effects of these atomic current loops are overcome if the material displays a net magnetic moment or has a long-range ordering of its magnetic moments [54-56]. All other types of magnetic behaviors are observed in materials that are at least partially attributed to unpaired electrons in their atomic shells, often in the 3*d *or 4*f *shells of each atom. Materials whose atomic magnetic moments are uncoupled display paramagnetism; thus, paramagnetic materials have moments with no long-range order, and there is a small positive magnetic susceptibility (*χ *≈ 0), e.g., pyrite [57-59]. Materials that possess ferromagnetism have aligned atomic magnetic moments of equal magnitude, and their crystalline structures allow for direct coupling interactions between the moments, which may strongly enhance the flux density (e.g., Fe, Ni, and Co). Furthermore, the aligned moments in ferromagnetic materials can confer a spontaneous magnetization in the absence of an applied magnetic field. Materials that retain permanent magnetization in the absence of an applied field are known as hard magnets. Materials having atomic magnetic moments of equal magnitude that are arranged in an antiparallel fashion display antiferromagnetism (e.g., troilite FeS). The exchange interaction couples the moments in such a way that they are antiparallel, therefore, leaving a zero net magnetization [60]. Above the Néel temperature, thermal energy is sufficient to cause the equal and oppositely aligned atomic moments to randomly fluctuate, leading to a disappearance of their long-range order. In this state, the materials exhibit paramagnetic behavior. Ferrimagnetism is a property exhibited by materials whose atoms or ions tend to assume an ordered but nonparallel arrangement in a zero applied field below a certain characteristic temperature known as the Néel temperature (e.g., Fe_3_O_4 _and Fe_3_S_4_). In the usual case, within a magnetic domain, a substantial net magnetization results from the antiparallel alignment of neighboring non-equivalent sublattices. The macroscopic behavior is similar to ferromagnetism. Above the Néel temperature, the substance becomes paramagnetic (Figure 2) [61,62].

## Applications

### Industrial applications

Magnetic iron oxides are commonly used as synthetic pigments in ceramics, paints, and porcelain. Magnetic encapsulates may find very important uses in many areas of life and also in various branches of industry. Such materials are interesting from both points of the fundamental study of materials science as well as their applications [63,64]. Hematite and magnetite have been applied as catalysts for a number of important reactions, such as the preparation of NH_3_, the desulfurization of natural gas, and the high-temperature water-gas shift reaction. Other reactions include the Fishere-Tropsch synthesis for hydrocarbons, the dehydrogenation of ethylbenzene to styrene, the oxidation of alcohols, and the large-scale synthesis of butadiene [65-67].

### Biomedical applications

Biomedical applications of magnetic nanoparticles can be classified according to their application inside or outside the body (*in vivo, in vitro*). For *in vitro *applications, the main use is in diagnostic separation, selection, and magnetorelaxometry, while for *in vivo *applications, it could be further separated in therapeutic (hyperthermia and drug-targeting) and diagnostic applications (nuclear magnetic resonance [NMR] imaging) [68-70].

### In vivo applications

Two major factors play an important role for the *in vivo *uses of these particles: size and surface functionality. Even without targeting surface ligands, superparamagnetic iron oxide NP [SPIOs] diameters greatly affect *in vivo *biodistribution. Particles with diameters of 10 to 40 nm including ultra-small SPIOs are important for prolonged blood circulation; they can cross capillary walls and are often phagocytosed by macrophages which traffic to the lymph nodes and bone marrow [71].

*1. Therapeutic applications*. Hyperthermia: Placing superparamagnetic iron oxide in altering current [AC] magnetic fields randomly flips the magnetization direction between the parallel and antiparallel orientations, allowing the transfer of magnetic energy to the particles in the form of heat, a property that can be used *in vivo *to increase the temperature of tumor tissues to destroy the pathological cells by hyperthermia. Tumor cells are more sensitive to a temperature increase than healthy ones [72,73]. In past studies, magnetite cationic liposomal nanoparticles and dextran-coated magnetite [74] have been shown to effectively increase the temperature of tumor cells for hyperthermia treatment in cell irradiation. This has been proposed to be one of the key approaches to successful cancer therapy in the future [75]. The advantage of magnetic hyperthermia is that it allows the heating to be restricted to the tumor area. Moreover, the use of subdomain magnetic particles (nanometer-sized) is preferred instead multidomain (micron-sized) particles because nanoparticles absorb much more power at tolerable AC magnetic fields [76,77] which is strongly dependent on the particle size and shape, and thus, having well-defined synthetic routes able to produce uniform particles is essential for a rigorous control in temperature.

*2. Drug delivery*. Drug targeting has emerged as one of the modern technologies for drug delivery. The possibilities for the application of iron oxide magnetic nanoparticles in drug targeting have drastically increased in recent years [78]. MNPs in combination with an external magnetic field and/or magnetizable implants allow the delivery of particles to the desired target area, fix them at the local site while the medication is released, and act locally (magnetic drug targeting) [79]. Transportation of drugs to a specific site can eliminate side effects and also reduce the dosage required. The surfaces of these particles are generally modified with organic polymers and inorganic metals or oxides to make them biocompatible and suitable for further functionalization by the attachment of various bioactive molecules [80,81]. The process of drug localization using magnetic delivery systems is based on the competition between the forces exerted on the particles by the blood compartment and the magnetic forces generated from the magnet.

3. Diagnostic applications

a. *NMR imaging*. The development of the NMR imaging technique for clinical diagnosis has prompted the need for a new class of pharmaceuticals, so-called magneto-pharmaceuticals. These drugs must be administered to a patient in order to (1) enhance the image contrast between the normal and diseased tissue and/or (2) indicate the status of organ functions or blood flow.

### *In vitro *applications

**Figure 3:**
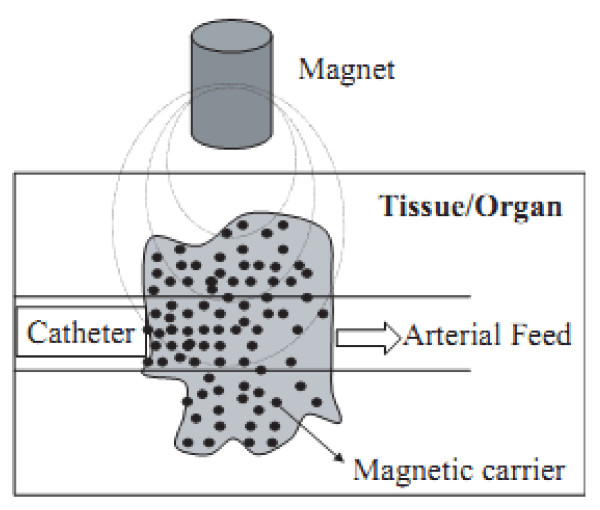
**Schematic representation of the magnetically driven transport of drugs to a specific region**. A catheter is inserted into an arterial feed to the tumor, and a magnetic stand is positioned over the targeted site.

1. *Diagnostic applications*

a. *Separation and selection*. At present, considerable attention is being paid to solid-phase extraction [SPE] as a way to isolate and preconcentrate desired components from a sample matrix. SPE is a routine extraction method for determining trace-level contaminants in environmental samples. Recently, nanometer-sized particles (nanoparticles, NPs) have gained rapid and substantial progress and have a significant impact on sample extraction. SPE offers an excellent alternative to the conventional sample concentration methods, such as liquid-liquid extraction [82-84]. The separation and preconcentration of the substance from large volumes of solution can be highly time consuming when using standard column SPE, and it is in this field where the use of magnetic or magnetizable adsorbents called magnetic solid-phase extraction [MSPE] gains importance. In this procedure, the magnetic adsorbent is added to a solution or suspension containing the target. This is adsorbed onto the magnetic adsorbent, and then, the adsorbent with the adsorbed target is recovered from the suspension using an appropriate magnetic separator. For separation and selection, the advantage of using magnetic nanoparticles instead magnetic microparticles is that we can prepare suspensions that are stable against sedimentation in the absence of an applied magnetic field. The applicability of iron oxide magnetic nanoparticles in MSPE is clearly evidenced by the fact that it already exists in the market companies (DYNAL Biotech) that commercialize these products (Figure 3).

b. *Magnetorelaxometry*. It was introduced as a method for the evaluation of immunoassays [85]. Magnetorelaxometry measures the magnetic viscosity, i.e., the relaxation of the net magnetic moment of a system of magnetic nanoparticles after removal of a magnetic field [86]. There are two different relaxation mechanisms. First, the internal magnetization vector of a nanoparticle relaxes in the direction of the easy axis inside the core; this is called Néel relaxation [87]. Second, particles accomplish rotational diffusion in a carrier liquid, called Brownian relaxation [88]. Néel and Brownian relaxation can be distinguished by their different relaxation times [89]. Furthermore, Brownian relaxation can take place only in liquids, whereas Néel relaxation does not depend on the dispersion of the nanoparticles. The fact that magnetorelaxometry depends on the core size, the hydrodynamic size, and the anisotropy allows this technique to distinguish between the free and bound conjugates by their different magnetic behavior and therefore can be used as an analytical tool for the evaluation of immunoassays [90]. For this application, the benefits of reducing the particle size to the nanometer size are similar to those described for separation and selection applications.

c. *Magnetic resonance imaging*. At the boundary between nanomaterials and medical diagnostics, superparamagnetic iron oxide NPs are proving to be a class of novel probes useful for *in vitro *and *in vivo *cellular and molecular imaging. Superparamagnetic contrast agents have an advantage of producing an enhanced proton relaxation in magnetic resonance imaging [MRI] in comparison with paramagnetic ones. Consequently, less amounts of a SPIO agent are needed to dose the human body than a paramagnetic one. To apply the magnetic fluids to a MRI contrast agent, a SPIO should be dispersed into a biocompatible and biodegradable carrier. Recently, Muller et al. [91] comprehensively reviewed the applications of super paramagnetic iron oxide NPs as a contrast agent. However, MRIs are not convenient for *in situ *monitoring. Thus, a sensitive and simple technique for *in situ *monitoring of the NPs in living cells is desirable. Compared to conventional organic fluorescence probes, advantages of the nanometer-sized fluorescence probes mainly include their higher photostability and stronger fluorescence. The main problem in cell imaging using the fluorescent nanoprobes is that the fluorescence signal is easily affected by the background noises caused by the cells, matrix, and the nonspecific scattering lights. The high signal to noise (S/N) ratio is not easy to obtain.

d. *Bioseparation*. In a biomedical study, separation of specific biological entities (e.g., DNAs, proteins, and cells) from their native environment is often required for analysis. Superparamagnetic colloids are ideal for this application because of their on-off nature of magnetization with and without an external magnetic field, enabling the transportation of biomaterials with a magnetic field. In a typical procedure for separation, the biological entities are labeled by superparamagnetic colloids and then subjected to separation by an external magnetic field [92]. Nanometer-sized magnetic particles, such as super paramagnetic iron oxide particles, have been extensively used for separation and purification of cells and biomolecules in bioprocesses [93-95]. Due to their small size and high surface area, MNPs have many superior characteristics for these bioseparation applications compared to those of the conventional micrometer-sized resins or beads such as good dispersability, the fast and effective binding of biomolecules, and reversible and controllable flocculation. One of the trends in this subject area is the magnetic separation using antibodies to provide highly accurate antibodies that can specifically bind to their matching antigens on the surface of the targeted species.

2. *Catalysis applications*. In recent years, catalysts supported by MNPs have been extensively used to improve the limitation of heterogeneous catalysis. Magnetically driven, separations make the recovery of catalysts in a liquid-phase reaction much easier than using cross flow filtration and centrifugation, especially when the catalysts are in the submicrometer size range. Such small and magnetically separable catalysts could combine the advantages of high dispersion and reactivity with easy separation. In terms of recycling expensive catalysts or ligands, immobilization of these active species on MNPs leads to the easy separation of catalysts in a quasi-homogeneous system [96]. The various types of transition metal-catalyzed reactions using catalytic sites grafted onto MNPs that have emerged recently include carbon-carbon cross-coupling reactions, hydroformylation [97], hydrogenation, and polymerization [98] reactions. Other reports on MNP-supported catalysts include enzymes for carboxylate resolution, amino acids for ester hydrolysis, and organic amine catalysts promoting Knoevenagel and related reactions.

### Environmental applications

A similarly important property of nanoscale iron particles is their huge flexibility for *in situ *applications. Modified iron nanoparticles, such as catalyzed and supported nanoparticles, have been synthesized to further enhance their speed and efficiency of remediation [99]. In spite of some still unresolved uncertainties associated with the application of iron nanoparticles, this material is being accepted as a versatile tool for the remediation of different types of contaminants in groundwater, soil, and air on both the experimental and field scales [100]. In recent years, other MNPs have been investigated for the removal of organic and inorganic pollutants.

### Organic pollutants

There are a few articles about the removal of high concentrations of organic compounds which are mostly related to the removal of dyes. The MNPs have a high capacity in the removal of high concentrations of organic compounds [101-103]. Dyes are present in the wastewater streams of many industrial sectors such as in dyeing, textile factories, tanneries, and in the paint industry. Therefore, the replacement of MNPs with an expensive or low efficient adsorbent for treatment of textile effluent can be a good platform which needs more detailed investigations.

### Inorganic pollutants

A very important aspect in metal toxin removal is the preparation of functionalized sorbents for affinity or selective removal of hazardous metal ions from complicated matrices. MNPs are used as sorbents for the removal of metal ions. Thus, MNPs show a high [104-106] capacity and efficiency in the removal of different metal ions due to their high surface area with respect to micron-sized sorbents. These findings can be used to design an appropriate adsorption treatment plan for the removal and recovery of metal ions from wastewaters.

### Analytical applications

1. *Fluorescence techniques*. Due to their small size, magnetic luminescent NPs offer a larger surface area-to-volume ratio than currently used microbeads, which result in a good reaction homogeneity and faster reaction kinetics. Thus, the preparation of magnetic fluorescent particles, such as polystyrene magnetic beads with entrapped organic dyes/quantum dots [QDs] or shells of QDs [107], iron oxide particles coated with dye-doped silica shells, and silica NPs embedded with iron oxide and QDs, is easier. However, their application is limited mostly to biological applications, such as cellular imaging. Only a few papers have reported the use of dual-functional NPs for multiplexed quantitative bioanalysis. The magnetic properties of the MLNPs allowed their manipulation by an external magnetic field without the need of centrifugation or filtration. Their optical characteristics (sharp emission, photostability, long lifetime) facilitated the implementation of an internal calibration in the detection system. This introduced a unique internal quality control and easy quantifications in the multiplexed immunoanalysis. This method developed and enables a direct, simple, and quantitative multiplex protein analysis using conventional organic dyes and can be applied for disease diagnostics and detection of biological threats.

1. Inorganic and hybrid coatings (or shells) on colloidal templates have been prepared by precipitation and surface reactions [108]. By adequate selection of the experimental conditions, mainly the nature of the precursors, temperature, and pH, this method can give uniform, smooth coatings, and therefore lead to monodispersed spherical composites. Using this technique, submicrometer-sized anionic polystyrene lattices have been coated with uniform layers of iron compounds [109] by aging at an elevated temperature and by dispersions of the polymer colloid in the presence of aqueous solutions of ferric chloride, urea, hydrochloric acid, and polyvinyl pyrrolidone. One of the most promising techniques for the production of superparamagnetic composites is the layer-by-layer self-assembly method. This method was firstly developed for the construction of ultrathin films [110] and was further developed by Caruso et al. [111] for the controlled synthesis of novel nanocomposite core-shell materials and hollow capsules. It consists of the stepwise adsorption of charged polymers or nanocolloids and oppositely charged polyelectrolytes onto flat surfaces or colloidal templates, exploiting primarily electrostatic interactions for layer buildup. Using this strategy, colloidal particles have been coated with alternating layers of polyelectrolytes, nanoparticles, and proteins [112]. Furthermore, Caruso et al. have demonstrated that submicrometer-sized hollow silica spheres or polymer capsules can be obtained after removal of the template from the solid-core multilayered-shell particles either by calcination or by chemical extraction. Their work in the preparation of iron oxide superparamagnetic and monodisperse, dense, and hollow spherical particles that could be used for biomedical applications deserves special mention.

2. *Encapsulation of magnetic nanoparticles in polymeric matrixes*. Encapsulation of inorganic particles into organic polymers endows the particles with important properties that bare uncoated particles lack [113]. Polymer coatings on particles enhance compatibility with organic ingredients, reduce susceptibility to leaching, and protect particle surfaces from oxidation. Consequently, encapsulation improves dispersibility, chemical stability, and reduces toxicity [114]. Polymer-coated magnetite nanoparticles have been synthesized by seed precipitation polymerization of methacrylic acid and hydroxyethyl methacrylate in the presence of the magnetite nanoparticles [115]. Cross-linking of polymers has also been reported as an adequate method for the encapsulation of magnetic nanoparticles. To prepare the composites by this method, first, mechanical energy needs to be supplied to create a dispersion of magnetite in the presence of aqueous albumin [116], chitosan [117], or PVA polymers [118]. More energy creates an emulsion of the magnetic particle sol in cottonseed [119], mineral [120], or vegetable oil [121]. Depending upon composition and reaction conditions, the addition of a cross-linker and heat results in a polydispersed magnetic latex, 0.3 microns in diameter, with up to 24 wt.% in magnetite content [122]. Recently, the preparation of superparamagnetic latex via inverse emulsion polymerization has been reported [123]. A 'double-hydrophilic' diblock copolymer, present during the precipitation of magnetic iron oxide, directs nucleation, controls growth, and sterically stabilizes the resulting 5-nm superparamagnetic iron oxide. After drying, the coated particles repeptize creating a ferrofluid-like dispersion. Inverse emulsification of the ferrofluid into decane, aided by small amounts of diblock copolymer emulsifier along with ultrasonication, creates mini droplets (180 nm) filled with magnetic particles and monomer. Subsequent polymerization generates magnetic latex. A novel approach to prepare superparamagnetic polymeric nanoparticles by synthesis of the magnetite core and polymeric shell in a single inverse microemulsion was reported by Chu et al. [124]. Stable magnetic nanoparticle dispersions with narrow size distribution were thus produced. The microemulsion seed copolymerization of methacrylic acid, hydroxyethyl methacrylate, and cross-linker resulted in a stable hydrophilic polymeric shell around the nanoparticles. Changing the monomer concentration and water/surfactant ratio controls the particle size.

*Encapsulation of magnetic nanoparticles in inorganic matrixes*. An appropriate tuning of the magnetic properties is essential for the potential use of the superparamagnetic composites. In this way, the use of inorganic matrixes, in particular of silica, as dispersion media of superparamagnetic nanocrystals has been reported to be an effective way to modulate the magnetic properties by a simple heating process [125]. Another advantage of having a surface enriched in silica is the presence of surface silanol groups that can easily react with alcohols and silane coupling agents [126] to produce dispersions that are not only stable in nonaqueous solvents, but also provide the ideal anchorage for covalent bonding of specific ligands. The strong binding makes desorption of these ligands a difficult task. In addition, the silica surface confers a high stability to suspensions of the particles at high volume fractions, changes in pH, or electrolyte concentration [127]. Recently, we have been successful in preparing submicronic silica-coated maghemite hollow and dense spheres with a high loading of magnetic material by aerosol pyrolysis [128]. Silica-coated γ-Fe_2_O_3 _hollow spherical particles with an average size of 150 nm were prepared by aerosol pyrolysis of methanol solutions containing iron ammonium citrate and tetraethoxysilane [TEOS] at a total salt concentration of 0.25 M [129]. During the first stage, the rapid evaporation of the methanol solvent favors the surface precipitation (i.e., formation of hollow spheres) of the components [130]. The low solubility of the iron ammonium citrate in methanol when compared with that of TEOS promotes the initial precipitation of the iron salt solid shell. During the second stage, the probable continuous shrinkage of this iron salt solid shell facilitates the enrichment at the surface of the silicon oxide precursor (TEOS). In the third stage, the thermal decomposition of precursors produces the silica-coated γ-Fe_2_O_3 _hollow spheres. The formation of the γ-Fe_2_O_3 _is associated with the presence of carbonaceous species coming from the decomposition of the methanol solvent and from the iron ammonium citrate and TEOS. On the other hand, the aerosol pyrolysis of iron nitrate and TEOS at a total salt concentration of 1 M produced silica-coated γ-Fe_2_O_3 _dense spherical particles with an average size of 250 nm. The increase in salt concentration to a value of 1 M favors the formation of dense spherical particles. Sedimentation studies of these particles have shown that they are particularly useful for separation applications [131]. A W/O microemulsion method has also been used for the preparation of silica-coated iron oxide nanoparticles [132]. Three different non-ionic surfactants (Triton X-100, Dow Chemical Company, Midland, MI, USA; Igepal CO-520, and Brij-97) have been used for the preparation of microemulsions, and their effects on the particle size, crystallinity, and the magnetic properties have been studied

3. The iron oxide nanoparticles are formed by the coprecipitation reaction of ferrous and ferric salts with inorganic bases. A strong base, NaOH, and a comparatively mild base, NH_4_OH, have been used with each surfactant to observe whether the basicity influences the crystallization process during particle formation. All these systems show magnetic behavior close to that of superparamagnetic materials. By using this method, magnetic nanoparticles as small as 1 to 2 nm and of very uniform size (standard deviation less than 10%) have been synthesized. A uniform silica coating as thin as 1 nm encapsulating the bare nanoparticles is formed by the base-catalyzed hydrolysis and the polymerization reaction of TEOS in the microemulsion. It is worth mentioning that the small particle size of the composite renders these particles a potential candidate for their use in *in vivo *applications.

### Size selection methods

Biomedical applications like magnetic resonance imaging, magnetic cell separation, or magnetorelaxometry control the magnetic properties of the nanoparticles in magnetic fluids. Furthermore, these applications also depend on the hydrodynamic size. Therefore, in many cases, only a small portion of particles contributes to the desired effect. The relative amount of the particles with the desired properties can be increased by the fractionation of magnetic fluids [133]. Common methods currently used for the fractionation of magnetic fluids are centrifugation [134] and size-exclusion chromatography [135]. All these methods separate the particles via nonmagnetic properties like density or size. Massart et al [136] have proposed a size sorting procedure based on the thermodynamic properties of aqueous dispersions of nanoparticles. The positive charge of the maghemite surface allows its dispersion in aqueous acidic solutions and the production of dispersions stabilized through electrostatic repulsions. By increasing the acid concentration (in the range 0.1 to 0.5 mol l-1*)*, interparticle repulsions are screened, and phase transitions are induced. Using this principle, these authors describe a two-step size sorting process in order to obtain significant amounts of nanometric monosized particles with diameters between typically 6 and 13 nm. As the surface of the latter is not modified by the size sorting process, usual procedures are used to disperse them in several aqueous or oil-based media. Preference should be given, however, to partitions based on the properties of interest, in this case, the magnetic properties. So far, magnetic methods have been used only for the separation of magnetic fluids, for example, to remove aggregates by magnetic filtration [137]. Recently, the fractionation of magnetic nanoparticles by flow field-flow fractionation was reported [138]. Field-flow fractionation is a family of analytical separation techniques [139,140], in which the separation is carried out in a flow with a parabolic profile running through a thin channel. An external field is applied at a right angle to force the particles toward the so-called accumulation wall [141,142].

## Competing interests

The authors declare that they have no competing interests.

## Authors' contributions

SD conceived of the study, and participated in its design and coordination. All authors read and approved the final manuscript. AA participated in the sequence alignment and drafted the manuscript.
